# A plugin for the Ensembl Variant Effect Predictor that uses MaxEntScan to predict variant spliceogenicity

**DOI:** 10.1093/bioinformatics/bty960

**Published:** 2018-11-23

**Authors:** Jannah Shamsani, Stephen H Kazakoff, Irina M Armean, Will McLaren, Michael T Parsons, Bryony A Thompson, Tracy A O’Mara, Sarah E Hunt, Nicola Waddell, Amanda B Spurdle

**Affiliations:** 1 Department of Genetics and Computational Biology, QIMR Berghofer Medical Research Institute, Brisbane QLD, Australia; 2 European Molecular Biology Laboratory, European Bioinformatics Institute, Wellcome Genome Campus, Hinxton, Cambridge, UK; 3 Centre for Epidemiology and Biostatistics, School of Population and Global Health, University of Melbourne, Melbourne VIC, Australia

## Abstract

**Summary:**

Assessing the pathogenicity of genetic variants can be a complex and challenging task. Spliceogenic variants, which alter mRNA splicing, may yield mature transcripts that encode non-functional protein products, an important predictor of Mendelian disease risk. However, most variant annotation tools do not adequately assess spliceogenicity outside the native splice site and thus the disease-causing potential of variants in other intronic and exonic regions is often overlooked. Here, we present a plugin for the Ensembl Variant Effect Predictor that packages MaxEntScan and extends its functionality to provide splice site predictions using a maximum entropy model. The plugin incorporates a sliding window algorithm to predict splice site loss or gain for any variant that overlaps a transcript feature. We also demonstrate the utility of the plugin by comparing our predictions to two mRNA splicing datasets containing several cancer-susceptibility genes.

**Availability and implementation:**

Source code is freely available under the Apache License, Version 2.0: https://github.com/Ensembl/VEP_plugins.

**Supplementary information:**

Supplementary data are available at *Bioinformatics* online.

## 1 Introduction

RNA splicing is a tightly-regulated process that involves the excision of non-coding intronic sequences from nascent precursor mRNA and the ligation of coding exons to produce mature transcripts ready for translation into protein. The splicing reaction is catalyzed by molecular machinery which recognizes short consensus sequences called donor and acceptor sites at the intron/exon boundaries. Variants that impact these sequences or other regulatory sequences may disrupt normal splicing and result in the synthesis of aberrant or non-functional transcript or protein products. The identification of such splicing defects remains a challenge, despite nearly a third of all pathogenic (disease-associated) variants being predicted to impact normal splicing (Lim *et al.*, 2011; Sterne-Weiler *et al.*, 2011). Variants that affect the highly conserved GT-AG dinucleotides in the native donor and acceptor splice sites are routinely assessed for spliceogenicity because they are generally presumed to cause severe splicing aberrations. However, variants outside of the native splice sites are often overlooked for their role in splicing. Although these are less likely to impact splicing, variants outside the native splice sites have been shown to abolish native splice sites and activate *de novo* or pre-existing cryptic splice sites (Houdayer *et al.*, 2012; Rodríguez-Balada *et al.*, 2016; Sanz *et al.*, 2010; Théry *et al.*, 2011; Walker *et al.*, 2010). To address this issue and enable the rapid assessment of complex sequence variants, we developed a plugin for the Ensembl Variant Effect Predictor (VEP) (McLaren *et al.*, 2016) that encapsulates MaxEntScan (Eng *et al.*, 2004; Yeo and Burge, 2004) and uses its functionality to generate splice prediction scores for any variant that overlaps a transcript feature. We demonstrate the utility of the plugin by comparing our spliceogenicity predictions to *in vitro* splicing results using the current score thresholds defined by the Evidence-based Network for the Interpretation of Germline Mutant Alleles (ENIGMA) consortium (Spurdle *et al.*, 2012).

## 2 Approach

The core functionality of the MaxEntScan plugin is designed to provide three sets of scores that can be incorporated into a comprehensive framework to predict the fitness of a given sequence motif as either a donor or an acceptor splice site based on a maximum entropy model. First, the plugin provides scores necessary to predict the loss of a native splice site for single nucleotide variants (SNVs). These are the scores for reference and alternate sequence motifs, using 9-mers for native donor splice sites and 23-mers for acceptor splice sites as described in (Yeo and Burge, 2004). Second, a sliding window algorithm called MES-SWA was added to assess deeper intronic, exonic and other types of variants, such as insertions and deletions (Vallée *et al.*, 2016). A scoring window is slid across the reference or alternate sequence such that the reference or alternate allele moves from either the 9th position (donor) or 23rd position (acceptor) to the 1st position to capture the highest score as the most fit potential donor or acceptor splice site. To assess the impact of variants, reference comparison scores are also provided. For SNVs, the reference comparison scores are derived from the sequence with the same frame as the highest scoring *k*-mer containing the alternate allele. For all other variants, the frame of the highest scoring *k*-mer containing the reference sequence is used to derive the comparison score. Third, the plugin provides additional scores to assess if a *de novo* donor or acceptor can out-compete the native splice site. This is achieved using the MES-NCSS function which scores the nearest canonical donor and acceptor splice sites both upstream and downstream of the variant.

## 3 Application examples–analysis of mRNA splicing datasets for cancer gene variants

**Fig. 1.**
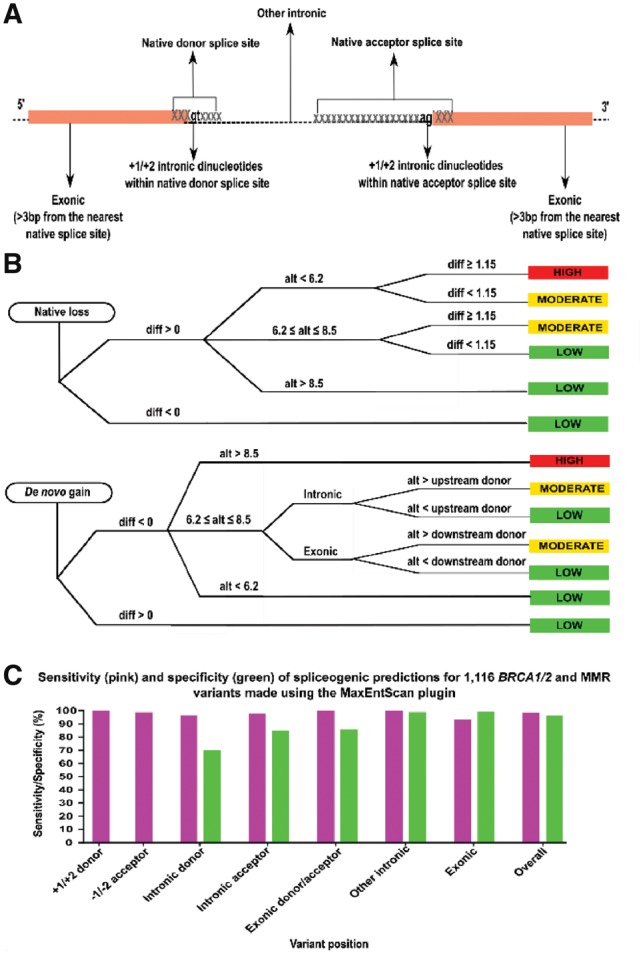
(**A**) The MaxEntScan plugin provides scores for sequence motifs within the native splice sites and other intronic and exonic regions. The native donor splice site is a 9-mer that overlaps the last three nucleotides of an exon and the first six nucleotides of a downstream intron. The native acceptor splice site is a 23-mer that overlaps the last 20 nucleotides of an intron and the first three nucleotides of a downstream exon. (**B**) Variants that overlapped the native splice sites were assessed for native splice site loss whilst variants outside of the native splice sites were assessed for gain of a *de novo* or cryptic splice site. Spliceogenicity was assessed using the reference (ref), alternate (alt) and difference (diff; ref–alt) maximum entropy scores and the ENIGMA score thresholds. SNVs within the native splice site were assessed for splice site loss using the native splice site scores, whilst indels that overlapped the native splice sites were assessed for splice site loss using the MES-SWA function. Variants predicted to diminish splicing (diff > 0) were further classified as having a high (alt < 6.2), moderate (6.2 ≤ alt ≤ 8.5) or low (alt > 8.5) potential of disrupting native splice sites. High and moderate classifications may also be downgraded to moderate and low, respectively (diff < 1.15). Creation of a *de novo* or cryptic splice site was assessed using the MES-SWA and MES-NCSS functions. Variants predicted to increase splicing (diff < 0) were further classified as having a high (alt > 8.5), moderate (6.2 ≤ alt ≤ 8.5) or low (alt < 6.2) potential of creating a *de novo* or cryptic splice site. Variants were only classified as having moderate potential if they could be shown to outcompete the nearest native splice site. (**C**) Sensitivity (pink) and specificity (green) for spliceogenic predictions of 1116 *BRCA1/2* and MMR variants made using the MaxEntScan plugin. Variants predicted having a high or moderate potential of native loss or *de novo* gain were expected to cause splicing aberrations. Spliceogenic predictions were compared to the reported *in vitro* splicing assays. Sensitivity measures the proportion of variants correctly predicted causing splicing aberrations, whilst specificity measures the proportion of variants correctly predicted to retain splicing profiles (100% reflects a perfect prediction). The specificity to predict normal splicing across the GT-AG donor and acceptor dinucleotides could not be calculated as only one true negative result was identified in those regions

We applied our plugin to 1116 variants in the *BRCA1* and *BRCA2* DNA damage repair genes and the *MLH1*, *MSH2*, *MSH6* and *PMS2* mismatch repair genes, which have been previously tested for possible splicing aberrations using mRNA assays. Data was collated from multiple publications and existing databases, summarized in Supplementary Table S1. An overview of the datasets is described in the Supplementary Material. The utility of this plugin to predict variant spliceogenicity was analyzed based on two conditions: loss of native splice sites, or gain of *de novo* splice site. Variants assessed in this analysis include SNVs, insertions and deletions within the native splice sites and other intronic and exonic regions (Fig. 1A).

The current ENIGMA thresholds (https://enigmaconsortium.org) were used to classify variant spliceogenicity depending on variant type and location (Fig. 1B). The sensitivity of this plugin to predict splicing aberrations across different regions varied between 93.3% and 100%, and the overall sensitivity to predict splicing aberrations reached 98.7%, whilst the overall specificity to predict normal splicing reached 96.5% (Fig. 1C;Supplementary Table S2). In summary, the spliceogenicity predictions using the ENIGMA thresholds compared well with the observed *in vitro* splicing results. Other user-defined thresholds may also be applied to assess variant spliceogenicity beyond the genes assessed here. The MaxEntScan plugin provides a simple and flexible means of assessing variant spliceogenicity, regardless of the location.

## Funding

This work was supported by a QIMR Berghofer PhD scholarship (J.S.). N.W. is supported by an National Health and Medical Research Council of Australia Senior Research Fellowship (APP1139071). A.B.S. is supported by an National Health and Medical Research Council of Australia Senior Research Fellowship (APP1061779). T.A.O.’M. is supported by an National Health and Medical Research Council of Australia Early Career Fellowship (APP1111246). Ensembl receives majority funding from the Wellcome Trust (grant number WT108749/Z/15/Z) with additional funding for specific project components from the National Human Genome Research Institute (U41HG007823 and 2U41HG007234), the Biotechnology and Biological Sciences Research Council (BB/N019563/1 and BB/M011615/1), Open Targets, the Wellcome Trust (WT104947/Z/14/Z, WT200990/Z/16/Z, WT201535/Z/16/Z, WT108749/Z/15/A, WT212925/Z/18/Z), ELIXIR: the research infrastructure for life-science data and the European Molecular Biology Laboratory. This project has received funding from the European Union’s Horizon 2020 research and innovation programme under grant agreement n° 733161 (MultipleMS).

*Conflict of Interest*: none declared.

## Supplementary Material

bty960_Supplementary_Data
