# Supplementary material for: A plugin for the Ensembl Variant Effect Predictor that uses MaxEntScan to predict variant spliceogenicity
Source: Bioinformatics. 2018 Nov 23;35(13):2315–7. doi: 10.1093/bioinformatics/bty960 (PMC6596880; doi:10.1093/bioinformatics/bty960)
Supplement: bty960_Supplementary_Data [file bty960_supplementary_data.zip › bty960-Suppl_data/Supplementary_document.doc]

**A plugin for the Ensembl Variant Effect Predictor that uses MaxEntScan to predict variant spliceogenicity**

Jannah Shamsani*^1^, Stephen H. Kazakoff*^1^, Irina M. Armean^3^, Will McLaren^3^, Michael T. Parsons^1^, Bryony A. Thompson^2^, Tracy A. O’Mara^1^, Sarah E. Hunt^3^, Nicola Waddell^1^ and Amanda B. Spurdle^1^ *Contributed equally

**Overview of datasets**

A total of 1,116 variants were assessed for their effect on mRNA splicing via the disruption or creation of donors/acceptor sites. VEP version 92.3 and plugin functionality released in version 94 were used to annotate the variant (Table S1). Two datasets were included in this analysis. The first dataset includes 601 previously published *BRCA1* and *BRCA2* variants with *in vitro* and/or *ex vivo* splicing evidence. A subset of these variants were previously used by the ENIGMA Consortium [10] as a training dataset to establish score thresholds for predicting splice aberrations <https://enigmaconsortium.org/library/general-documents/enigma-classification-criteria/>. The second dataset was included to test the ENIGMA score thresholds, and is composed of 596 published and unpublished variants in the MMR genes (*MLH1*, *MSH2*, *MSH6* and *PMS2*) with reported *in vitro* and/or *ex vivo* splicing evidence. This information on splicing evidence was collated from the literature and/or the databases hosted by UMD ([www.umd.be](http://www.umd.be/); MLH1, MSH2, MSH6 databases) or the InSiGHT consortium (<https://www.insight-group.org/variants/databases/>). Performance analysis excluded 68 *in vitro* splicing aberrations presumed to be caused by alteration of exonic splicing enhancers or silencers, because the MaxEntScan algorithm is not designed to predict these types of splicing aberrations. Another nine reported “aberrations” that have since shown to be naturally occurring isoforms, and four variants with low quality results making the splicing evidence uninterpretable, were also disregarded.

**Table S2: Spliceogenicity prediction compared to the reported mRNA splicing aberrations**

| **Variant location** | **Type of reported splicing aberration** | **# of reported variant (*in vitro* aberration)** | **# of variant (MaxEntScan predicted high aberration)** | **# of variant (MaxEntScan predicted moderate aberration)** | **# of variant (MaxEntScan predicted no effect)** | **TP** | **TN** | **FP** | **FN** | **Specificity (%)^≠^** | **Sensitivity (%)^¥^** |
| --- | --- | --- | --- | --- | --- | --- | --- | --- | --- | --- | --- |
| +1/+2 intronic donor | donor loss | 90 | 89 | 1 | 0 | 90 | - | - | 0 | - | 100 |
|  | no aberration | 1 | 1 | 0 | 0 | - | 0 | 1 | - |  |  |
| -1/-2 intronic acceptor | acceptor loss | 81 | 80 | 0 | 1 | 80 | - | - | 1 | - | 98.8 |
|  | no aberration | 1 | 1 | 0 | 0 | - | 0 | 1 | - |  |  |
| Intronic donor  [+3 to +6] | donor loss | 58 | 52 | 4 | 2 | 56 | - | - | 2 | 70.0 | 96.6 |
|  | no aberration | 10 | 1 | 2 | 7 | - | 7 | 3 | - |  |  |
| Intronic acceptor  [-3 to -20] | acceptor loss | 52 | 44 | 7 | 1 | 51 | - | - | 1 | 84.9 | 98.1 |
|  | no aberration | 73 | 2 | 9 | 62 | - | 62 | 11 | - |  |  |
| Exonic  [ ≤ 3bp from native acceptor/donor] | donor/acceptor loss | 82 | 62 | 20 | 0 | 82 | - | - | 0 | 85.7 | 100 |
|  | no aberration | 28 | 1 | 3 | 24 | - | 24 | 4 | - |  |  |
| Other intronic | donor gain | 2 | 2 | 0 | 0 | 2 | - | - | 0 | 98.9 | 100 |
|  | no aberration | 90 | 1 | 0 | 89 | - | 89 | 1 | - |  |  |
| Exonic  [ > 3bp from native acceptor/donor] | donor gain | 12 | 9 | 3 | 0 | 12 | - | - | 0 | 99.1 | 93.3 |
|  | acceptor gain | 3 | 0 | 2 | 1 | 2 | - | - | 1 |  |  |
|  | no aberration | 533 | 5 | 0 | 528 | - | 528 | 5 | - |  |  |
| Overall | aberration | 380 | 338 | 37 | 5 | 375 | - | - | 5 | 96.5 | 98.7 |
|  | no aberration | 736 | 12 | 14 | 710 | - | 710 | 26 | - |  |  |
| TP: True positive; TN: True negative; FP: False positive; FN: False negative  ^≠^Specificity (True negative rate) = (TN/ (TN + FP))*100  ^¥^Sensitivity (True positive rate) = (TP/ (TP + FN))*100 | | | | | | | | | | | |
